# The safety and efficacy of through-and-through wire technique for ureteral Double-J stent placement

**DOI:** 10.12669/pjms.40.9.9424

**Published:** 2024-10

**Authors:** Cheng Shi Chen, Chu Hui Zeng, Ji Hoon Shin, Suyoung Park, Hai Liang Li, Fang Kun Li

**Affiliations:** 1 Cheng Shi Chen, MD Department of Radiology, Department of Medical Imaging, Changsha Medical University, Changsha, China; 2 Chu Hui Zeng, PhD Department of Radiology and Research Institute of Radiology, Department of Radiology and Research Institute of Radiology, Asan Medical Center, University of Ulsan College of Medicine, Seoul, South Korea; 3 Ji Hoon Shin, MD Department of Radiology, Department of Radiology and Research Institute of Radiology, The Affiliated Cancer Hospital of Zhengzhou University, Henan Cancer Hospital, Zhengzhou, China; 4 Suyoung Park, MD Department of Radiology, Gil Medical Center, Gachon University College of Medicine, Incheon, Republic of Korea; 5 Hai Liang Li, MD Department of Radiology, The Affiliated Cancer Hospital of Zhengzhou University, Henan Cancer Hospital, Zhengzhou, China; 6 Fang Kun Li, MD Department of Radiology, The Affiliated Cancer Hospital of Zhengzhou University, Henan Cancer Hospital, Zhengzhou, China

**Keywords:** Percutaneous nephrostomy, Double-J stent, Through-and-through wire technique

## Abstract

**Objective:**

To evaluate the efficacy and safety of the through-and-through wire (TTW) technique for antegrade ureteral Double-J stent placement after failure of either antegrade or retrograde ureteral stent placement.

**Method:**

This retrospective study analyzed the medical records of consecutive patients who underwent Double-J stent placement with the TTW technique at Asan Medical Center and Gil Medical Center between January 2016 and February 2023. Patient histories, reasons for employing the TTW technique, TTW pathways, and complications were reviewed. Eight patients were included in the study. The reasons for using the TTW technique were failure to advance a larger-diameter catheter, balloon catheter, or Double-J stent passing over the guidewire beyond the stricture (6/8, 75.0%); failure to negotiate the stricture with a guidewire (1/8, 12.5%); and guidewire passing through a ureteropelvic junction defect (1/8, 12.5%).

**Results:**

TTW was applied either between a percutaneous nephrostomy (PCN) and the urethral orifice (n=4), between a PCN and an ileostomy pouch (n=3), or between a left and right PCN (n = 1). Urologic assistance was required for retrograde ureteral cannulation in one male patient (12.5%). Subsequently, balloon dilation and/or Double-J stent placement were performed in all eight patients, resulting in 100% technical success. No major or minor complications occurred.

**Conclusions:**

The TTW technique was safe and effective in the undertaking of PCN and antegrade Double-J stent placement in patients for whom either antegrade or retrograde access had failed.

## INTRODUCTION

Double-J (DJ) stent placement is a routine procedure that assists urine drainage when a stricture or obstruction is present in the urinary tract. DJ stents facilitate the healing of the urinary tract and divert urine away from areas of leakage.^1^ Cystoscopic retrograde placement and percutaneous antegrade placement are two common approaches to DJ stent insertion, with success rates of 10-40% and 90%, respectively.^2^ Although the success rate associated with antegrade placement is much higher, the retrograde approach is usually preferred because it avoids percutaneous nephrostomy (PCN), which is inconvenient to maintain and can lead to bleeding or infection.^3^ However, in the cases of failed retrograde ureteral cannulation, the antegrade approach is usually attempted to complete the treatment.

When a stricture, leakage, or tortuosity is present, guidewire negotiation assisted by a catheter, regardless of the approach (retrograde or antegrade), may fail to pass through a certain point, and the guidewire may kink along the course of the ureter. In such difficult cases, the through-and-though wire (TTW) technique, which involves tightening both ends of the guidewire to gain sufficient support to straighten bends and kinks, has been proposed to facilitate the procedure.^4^,^5^ Given the TTW technique’s usefulness in vascular interventions but infrequent application in non-vascular procedures, as well as a lack of detailed descriptions of indications for the TTW technique,^6^-^8^ this study aimed to evaluate the efficacy and safety of DJ stent placement using the TTW technique. Other associated technical details were also investigated.

## METHODS

Medical records of consecutive patients who underwent PCN and antegrade DJ stent placement with the TTW technique between January 2016 and February 2023 were reviewed.Information about patient history, reasons for employing the TTW technique, TTW pathways, and complications was obtained. All patients provided written informed consent to undergo the procedures, which were performed in accordance with the Declaration of Helsinki and its revisions.

### Ethical Approval:

This study was approved by the institutional review boards of the two participating tertiary referral centers, Asan Medical Center (Approval number: S2023-0717-0001) and Gil Medical Center (Approval number: GDIRB2024-029), and the requirement for written informed consent was waived because of the retrospective design of the study.

The baseline characteristics of the patients included are listed in Table-I.. Eight patients (male/female: 4/4; mean age, 50.9 ± 18.1 years [range, 30-82 years]) who underwent DJ stent placement with the TTW technique were included. Although Patient #3 underwent bilateral DJ stent placement during a single-session procedure for bilateral ureteropelvic junction (UPJ)–ileal ureteral anastomotic strictures, the TTW technique was applied to the right side only, and antegrade DJ stent placement was performed on the left side. Thus, only the right side was included for further analysis. All patients other than Patient #2 (7/8; 87.5%) underwent open surgery for urogenital disorders. Underlying lesions or abnormalities were ureteral or anastomotic stricture (4/8; 50.0%), combined anastomotic stricture and tortuous ileal conduit (2/8; 25.0%), ureteral UPJ leakage (1/8; 12.5%), and tortuous ileal conduit (1/8; 12.5%).

**Table-I:** Baseline characteristics and procedural details.

#/ Sex/ Age	History	Abnormalities	Reason for TTW	TTW pathway	Detailed Technique
1/ M/ 38	Left renal cell carcinoma; partial nephrectomy and RFA	Left UPJ leakage	GW exited the ureter at the UPJ	PCN–urethral orifice	0.035” GW caught by a snare (15 mm) in the urinoma cavity near UPJ
2/ F/ 44	Fibromatosis and underlying horseshoe kidney	Right proximal ureteral stricture	5F catheter failed to negotiate the stricture over the inserted MGW	PCN–urethral orifice	8F long sheath–assisted 0.018” MGW caught by a snare (20 mm) in the UB/ 4-, 6-, 8-mm BD
3/ F/ 37	Cervical cancer; bilateral ileal ureter replacement	Bilateral UPJ-ileal ureteral anastomotic stricture	GW and MGW failed to negotiate the stricture	Right PCN–left PCN (right-side TTW only)	0.035” GW caught by a snare (15 mm) in the right renal pelvis/ 6-mm BD
4/ F/ 48	Endometriosis; laparoscopic surgery	Right distal ureteral stricture	5F catheter failed to negotiate the stricture over the inserted MGW	PCN–urethral orifice	8F long sheath–assisted 0.018” MGW caught by a snare (15 mm) in the UB/ 6-mm BD
5/ M/ 72	Bladder cancer; radical cystectomy and ileal conduit	Right uretero-ileal anastomotic stricture and tortuous ileal conduit	Balloon or DJ stent failed to negotiate the stricture over the inserted GW	PCN–ileostomy pouch	8F long sheath–assisted 0.035” GW caught by mosquito forceps at the ileostomy pouch/ 7-mm BD
6/ M/ 30	Bladder cancer; kidney transplant, radical cystectomy, and ileal conduit	Tortuous ileal conduit	Balloon or DJ stent failed to negotiate the tortuosity over the inserted GW	PCN–ileostomy pouch	0.035” GW caught by mosquito forceps at the ileostomy pouch/ 7-mm BD
7/ M/ 56	Chronic renal failure; right kidney transplant	Right proximal ureteral stricture	5F catheter failed to negotiate the stricture over the inserted MGW	PCN–urethral orifice	8F long sheath–assisted 0.018” GW caught by a snare (20 mm)/ 3-, 5-mm BD
8/ F/ 82	Bladder cancer; radical cystectomy and ileal conduit	Left uretero-ileal anastomotic stricture and tortuous ileal conduit	Balloon or DJ stent failed to negotiate the tortuosity over the inserted GW	PCN–ileostomy pouch	0.035” GW caught by mosquito forceps at the ileostomy pouch/ 7-mm BD

***Note:*** TTW, through-and-through wire; RFA, radiofrequency ablation; UPJ, ureteropelvic junction; GW, guidewire; PCN, percutaneous nephrostomy; MGW, microguidewire; UB, urinary bladder; BD, balloon dilation; DJ, Double-J.

### Indications:

The TTW technique was implemented if: The guidewire failed to pass the stricture or tortuosity, or The guidewire could pass the stricture or tortuosity, but a larger-diameter catheter, balloon catheter, or DJ stent failed to follow regardless of the underlying conditions causing it.

### Procedure:

After the retrograde approach failed, the antegrade approach was attempted under local anesthesia. Usually, patients were laid prone – as this position provided a significantly shorter nephrostomy tract length and more potential access sites that make percutaneous renal access easier and safer^9^ – to expose the costovertebral angle area during PCN and turned into supine for retrograde access.^2^ Those with a history of ileal conduit surgery or kidney transplantation were laid supine.

After the calyces of the renal collecting system were percutaneously punctured under sonographic and fluoroscopic guidance, a 0.018-inch short guidewire was inserted, followed by the exchange of a 5F sheath (Pinnacle TIF Tip, Terumo, Tokyo, Japan). Then, an attempt was made to pass a 0.035-inch hydrophilic stiff guidewire (Radifocus Guide Wire, Terumo, Tokyo, Japan) through the ureter into the bladder or stoma. If this attempt failed, then an 8F long sheath (Flexor Balkin Guiding Sheath, Cook, Bloomington, IN, USA) was advanced to assist the passage of the guidewire.^10^ If this strategy also failed, then a 0.018-inch microguidewire and a microcatheter were exchanged through the long sheath to pass the obstruction.

In the cases of failed antegrade access, a snare (Amplatz Goose Neck Snare; Plymouth, MN, USA) or mosquito forceps was inserted from the urethral orifice or stoma to catch the tip of the guidewire and pull it outside the body. Then, both ends of the guidewire were stretched firmly to increase the tensile strength and support the insertion of the balloon catheter (Mustang 0.035” Balloon Dilatation Catheter; Boston Scientific, Natick, MA, USA) or DJ stent (Flexima Ureteral Stent; Boston Scientific, Natick, MA, USA). For cases wherein a microguidewire was manually stretched, the microcatheter was first exchanged with a 5F catheter over the microguidewire. Then, the microguidewire was exchanged with a 0.035-inch guidewire through the 5F catheter for balloon dilation if a stricture was identified, followed by stent placement. A ureterogram was performed to confirm the position of the DJ stent without major urine leaks.

**Fig.1:**
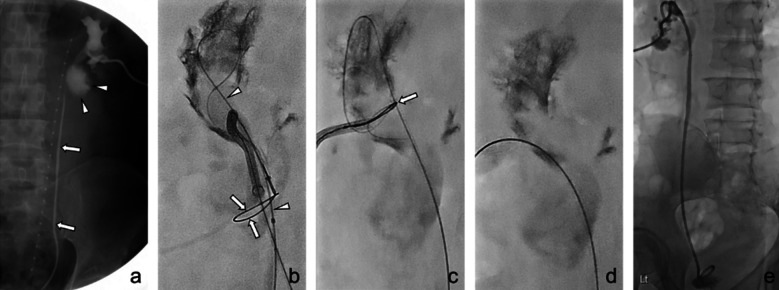
A 38-year-old man (Patient #1) with left ureteropelvic junction (UPJ) leakage after radiofrequency ablation for renal cell carcinoma. (a) After failed antegrade negotiation of the guidewire through the UPJ, an end-hole catheter (arrows) was inserted by a urologist for the through-and-through (TTW) wire technique. An antegrade pyelogram showed contrast leakage (arrowheads) at the UPJ. (b) An attempt was made to enter the open snare (arrows, inserted through a percutaneous nephrostomy [PCN]) with a guidewire (arrowheads) passed through the end-hole catheter. (c) The guidewire was confirmed to be caught by the snare (arrow). (d) The guidewire was externalized from the PCN to implement the TTW technique. (e) Finally, a Double-J stent was inserted after ureteral balloon dilation.

The detailed technical steps of the TTW technique for ureteral leakage after radiofrequency ablation are shown in Fig.1. After contrast extravasation was identified by pyelography, DJ stent placement was planned to restore ureteral patency. A 0.035-inch guidewire was used, but the guidewire passed through the ureteral leakage site. To avoid further injuries, a snare was inserted from the urethral orifice to catch the tip of the guidewire in the urinoma cavity near the UPJ, and the procedure was then successfully completed.

### Definition and follow-up:

Technical success was defined as accurate DJ stent placement using the TTW technique. The stent was changed every three to six months, either routinely or in cases of stent malfunction. According to the quality improvement guidelines established by the Society of Interventional Radiology, major complications were defined as those necessitating further treatment or prolonged hospitalization, and minor complications were those that resolved spontaneously.^11^

## RESULTS

The TTW technique was successfully performed for all eight patients, and an overall success rate of 100% was achieved. No procedure-related complications, such as pain and urinary tract infections, were reported after the procedure. During follow-up periods ranging from 1 week to 60 months, all the DJ stents were removed or exchanged where necessary. Procedural details are summarized in Table-I. An 8F long sheath was used in four patients (Patients #2, 4, 5, and 7) but failed to facilitate the guidewires passage.

The indications for implementing the TTW technique were failure to advance a larger-diameter catheter, balloon catheter, or DJ stent over the inserted guidewire (6/8; 75.0%; Patients #2, 4, 5, 6, 7, and 8), failure to negotiate the stricture with a guidewire (1/8, 12.5%; Patient #3), and the guidewire extending through a UPJ defect (1/8, 12.5%; Patient #1). The rendezvous location was in the urinary bladder (n = 3), ileostomy pouch (n = 3), urinoma cavity near the ureteropelvic junction (n = 1), or the contralateral renal pelvis (n = 1). Retrograde ureteral cannulation was performed by inserting a guidewire with cystoscopic assistance by a urologist in one male patient (Patient #1; 12.5%). After TTW technique implementation, DJ stents were respectively placed in four native ureters (4/8; 50.0%), one ileal ureter (1/8; 12.5%), and three ileal conduits (3/8; 37.5%).

**Fig.2:**
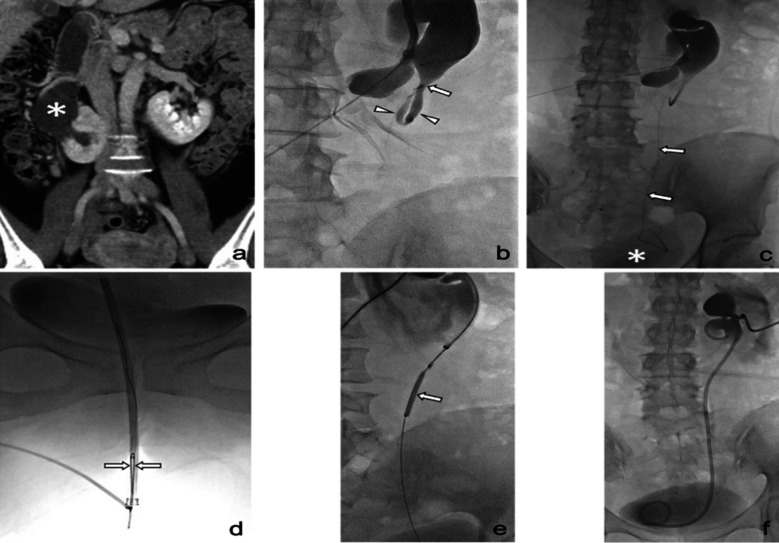
A 44-year-old woman (Patient #2) with underlying horseshoe kidney and fibromatosis underwent Double-J stent placement with the through-and-through wire technique. (a) Coronal contrast-enhanced computed tomography showed right hydronephrosis (asterisk) involving right ureteropelvic junction (UPJ) obstruction. (b) An antegrade pyelogram with contrast medium injected through a 5F catheter showed stenosis of the UPJ (arrow) and a tortuous proximal ureter (arrowheads). (c) After failed stricture negotiation using a 0.035-inch guidewire, there was successful passage of a 0.018-inch microguidewire (arrows) inserted through a 2F microcatheter, through the stricture, and into the urinary bladder (asterisk). (d) The tip of the microguidewire was captured by a snare (arrows) through a 9F sheath in the urinary bladder and withdrawn out of the urethra. (e) After the microguidewire was substituted with a 0.035-inch guidewire (not shown), serial 4-, 6-, and 8-mm balloon dilation (arrow) was performed at the stricture. (f) An 8F Double-J stent was successfully placed.

Among all cases described in the current study, the TTW technique was applied in four ureteral stricture cases, and a representative case is depicted in Fig.2. Depending on the severity of the stricture, a 0.035-inch guidewire was only able to pass the stricture in one case (1/4; 25.0%), and a 0.018-inch microguidewire was needed in the remaining three cases (3/4; 75.0%). This included Patient #3 who, with bilateral UPJ–ileal ureteral anastomotic strictures, represented a special case because the guidewire exited through the left PCN access instead of the urethral orifice. Since a left PCN was needed for DJ stent placement, the guidewire was externalized through this opening to reduce patient discomfort. Single or serial balloon dilation was performed to expand the stricture, followed by DJ stent placement.

**Fig.3:**
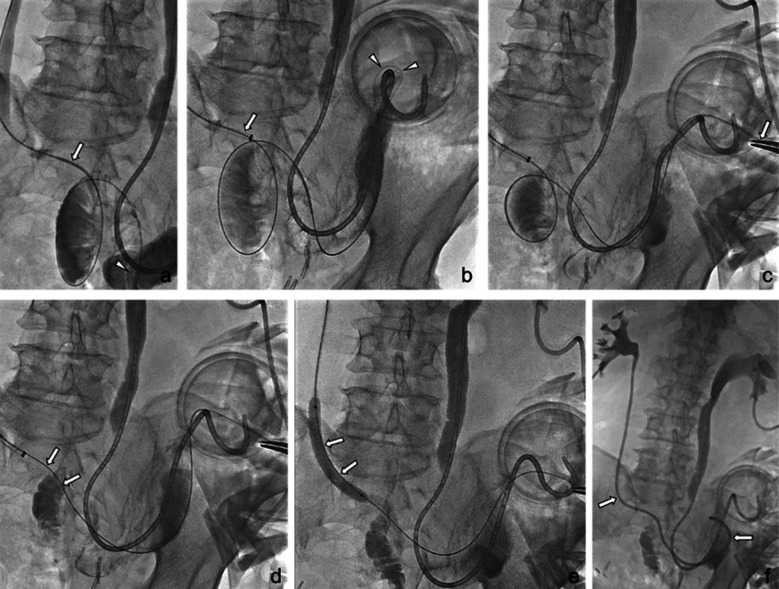
A 72-year-old man (Patient #5) with radical cystectomy and ileal conduit reconstruction for bladder cancer. (a) During negotiation of a 0.035-inch guidewire with a 5F catheter (arrow) from the left nephrostomy, the tip of the guidewire (arrowhead) could not reach the stoma due to stricture and looping of the anastomosis and ileal conduit. The right-sided ureteral stent was in situ. (b) With the use of a 7F Balkin sheath (arrow), the guidewire (arrowheads) could reach the stoma. (c) The tip of the guidewire was caught with a mosquito forceps (arrow) to execute the through-and-through technique. (d) Then, the guidewire (arrows) could be straightened. (e, f) 7-mm balloon dilation (arrows in e) was performed, followed by right ureteral stent insertion (arrows in f).

In addition to leakage and stricture formation, the TTW technique was also successfully performed in three cases of tortuous ileal conduit. In Patient #5, a 0.035-inch guidewire passed through the ureter in an antegrade direction, but it failed to negotiate the tortuosity in the ileal conduit (Fig.3). Since the guidewire was close to the ileostomy pouch, a snare was not necessary, and the procedure was completed using mosquito forceps.

## DISCUSSION

This study retrospectively analyzed the efficacy and safety of placing DJ stents using the TTW technique after the failure of retrograde and antegrade approaches regardless of underlying ureteral lesions. Overall, each of the eight patients successfully received a unilateral DJ stent placed using the TTW technique, meaning that the technical success rate was 100% in this study. No procedure-related complications were identified, demonstrating the efficacy and safety of the TTW technique for placing DJ stents.

Despite a much higher success rate associated with antegrade DJ stent placement over retrograde placement, the latter is generally more tolerable and acceptable to patients. Most of the time, retrograde placement can be done with the patient in a supine position, whereas the antegrade approach involving PCN requires most patients to lay prone, except for those with lesions on the front (e.g., ileal conduits). Additionally, the retrograde approach enters the urethral orifice or ileostomy pouch, which is a natural or artificial opening to the outside of the body.

However, for the antegrade approach, an opening for the guidewire and catheter needs to be created during the PCN step, which may carry a risk of bleeding or infection if the opening is not taken care of properly after the procedure. These points explain why the retrograde approach is usually preferred over the antegrade approach. In cases where neither of these approaches works, the combined antegrade and retrograde approach can be attempted.^12^

For successful DJ stent placement, smooth ureteral cannulation is the first and most critical step. When a stricture, leakage, or marked tortuosity forms whether iatrogenically or not,^13^ kinking or bending of the guidewire may occur, making it less likely for the guidewire and catheter to pass through. Although attempts can be made with smaller guidewires and catheters or a stiff guidewire, a false tract may be created in this scenario, increasing the risk of procedure-related complications.^14^ Another possible measure proposed by Chen *et al*. demonstrated the usefulness of long sheaths in stricture negotiation; however, the long sheath used in the present study did not solve this problem.^10^ Therefore, rather than pushing the guidewire or long sheath forcefully through the stricture, catching the tip of the guidewire from the other side might be a solution.

The combined antegrade/retrograde approach was first reported by Bagley *et al*. in 1985 for treating obliterated UPJ,^15^ and the concept of the rendezvous procedure was first introduced by Watson *et al*. in 2002.^16^ Since then, there formed two types of rendezvous procedures:

Endoscopic rendezvous that combines antegrade flexible ureteroendoscopic and retrograde semi-rigid ureteroscopic approaches; and combined endoscopic and radiologic approaches, consisting of retrograde cystoscopic insertion of a guidewire and antegrade access under fluoroscopy. In recent years, the latter approach has been more commonly used for complex ureteral stricture.^17^ For both techniques, the rendezvous technique requires the participation of physicians from other specialties (e.g., endoscopists and urologists) and their specialized devices (e.g., cystoscope).

When TTW was planned ahead for certain scenarios, for instance, difficult cases, scheduling and coordinating across departments are likely involved, possibly delaying the procedure for days or weeks, unintentionally elevating the risk of increased patient discomfort and infection. In addition, although the use of endoscopes provides more direct visualization of the inner structures, the larger bore of the instruments than interventional radiology devices can pose the main disadvantage of the procedure.^12^ In this present study, for female patients, urethral access was successfully achieved independently by interventional radiologists with an additional set of snare and sheath under fluoroscopic guidance.^18^ For male patients whose urethras are longer which, in general, makes intervention more challenging, independent completion of the TTW technique can be operator-dependent in certain cases.

For patients with ileostomy pouches, the end of the guidewire can be caught with forceps in the pouch outside the ileostomy opening under direct visualization. Overall, in most cases presented in this study, the procedure can be independently completed by interventional radiologists using the TTW technique, and this technique may represent the third and potentially the simplest form of rendezvous procedure for complex cases.

After establishing ureteral cannulation and externalizing the tip of the guidewire through the urethral orifice or ileostomy pouch, both ends of the guidewire can be tightly stretched to straighten bends or kinks inside the body. With firm fixation of the guidewire in place, dislodgement of the catheter or stent can be effectively prevented, and sufficient support can be quickly gained to allow the passage of the balloon and DJ stent through the curves. The TTW technique is believed to possess a few features. First, ureteral lesions can be quickly negotiated even in the context of tortuous structures like ileal conduits, especially by skilled operators with rich experience using snares.^19^,^20^ Second, the spectrum of instruments utilized is wide, and the device control techniques can be tailored according to the actual condition.^21^ Since the key is to catch the tip of the guidewire and bring it through the lesion, any device that can catch the tip should work.

Third, in select patients, such as women or patients with ileostomy pouches, the TTW technique can be completed independently by interventional radiologists, which simplifies scheduling and minimizes wait time, and patients whose antegrade stent placements fail can immediately proceed to the TTW technique using the same PCN access without being delayed to another day. Lastly, the TTW technique presents a versatile solution to strictures of various etiologies, limiting the use of surgery.^22^

### Limitations:

First, although the technical success rate was 100%, this study was a retrospective analysis of an uncommon but challenging procedure. Thus, it is difficult to compare the data, such as the success rate and procedure-related complications, with other techniques. Second, since most DJ stents were placed either retrogradely or antegradely, the sample size was small, and serum creatinine levels were not assessed. However, follow-up for up to 60 months demonstrated the successful implementation of this technique. Subsequent studies with larger sample sizes are warranted to further verify the safety and efficacy of the TTW technique.

## CONCLUSION

The TTW technique was safe and effective for DJ stent placement for various underlying ureteral lesions. This technique is indicated when stricture negotiation with a guidewire fails, subsequently leading to failed catheter or ureteral stent passage over a guidewire.

### Authors Contribution:

**CSC**, **CHZ**, and **JHS:** Conceived and designed, and review

**CSC**, **CHZ**, **SP**, **HLL** and **FKL:** Did literature search. Critical review.

**CHZ** and **JHS:** Did review, editing of manuscript.

**JHS:** Did review and final approval of manuscript.
